# Characteristic dynamic functional connectivity during sevoflurane-induced general anesthesia

**DOI:** 10.1038/s41598-023-43832-1

**Published:** 2023-11-29

**Authors:** Jingya Miao, Mohamed Tantawi, Mahdi Alizadeh, Sara Thalheimer, Faezeh Vedaei, Victor Romo, Feroze B. Mohamed, Chengyuan Wu

**Affiliations:** 1 Department of Neurosurgery and Radiology, Thomas Jefferson University, Philadelphia, PA USA; 2 Integrated Magnetic Resonance Imaging Center, Thomas Jefferson University, Philadelphia, PA USA; 3 Department of Anesthesia, Thomas Jefferson University, Philadelphia, PA USA

**Keywords:** Computational neuroscience, Neural circuits, Neuroscience, Network models

## Abstract

General anesthesia (GA) during surgery is commonly maintained by inhalational sevoflurane. Previous resting state functional MRI (rs-fMRI) studies have demonstrated suppressed functional connectivity (FC) of the entire brain networks, especially the default mode networks, transitioning from the awake to GA condition. However, accuracy and reliability were limited by previous administration methods (e.g. face mask) and short rs-fMRI scans. Therefore, in this study, a clinical scenario of epilepsy patients undergoing laser interstitial thermal therapy was leveraged to acquire 15 min of rs-fMRI while under general endotracheal anesthesia to maximize the accuracy of sevoflurane level. Nine recruited patients had fMRI acquired during awake and under GA, of which seven were included in both static and dynamic FC analyses. Group independent component analysis and a sliding-window method followed by k-means clustering were applied to identify four dynamic brain states, which characterized subtypes of FC patterns. Our results showed that a low-FC brain state was characteristic of the GA condition as a single featuring state during the entire rs-fMRI session; In contrast, the awake condition exhibited frequent fluctuations between three distinct brain states, one of which was a highly synchronized brain state not seen in GA. In conclusion, our study revealed remarkable dynamic connectivity changes from awake to GA condition and demonstrated the advantages of dynamic FC analysis for future studies in the assessments of the effects of GA on brain functional activities.

## Introduction

General anesthesia is a state of medically induced unconsciousness, during which the brain loses its capability to respond to external stimuli or to execute tasks. Sevoflurane, a halogenated inhalational anesthetic, is one of the commonly used agents during surgery. Although the mechanism by which sevoflurane affects neural communications remains unclear, a current working hypothesis is that it alters the connections between certain brain regions necessary for wakefulness^1,2^. Hence, identification of such alterations may help reveal the effects of sevoflurane on cognitive functions; as well as improve our understanding of the underlying brain architecture for consciousness^1^.

For assessments of intrinsic functional activity under anesthesia, resting state functional magnetic resonance imaging (rs-fMRI) has gained in popularity, as it allows for direct comparisons to the awake state without task-related external stimuli. In contrast to modalities such as electroencephalography (EEG) and positron emission tomography (PET), rs-fMRI provides high spatial resolution in the whole brain (both cerebral and subcortical areas) and avoids confounding effects of tracers^3–5^. Previous human rs-fMRI studies detected a variety of signal reductions under sevoflurane-induced anesthesia, notably: a decrease in BOLD signal amplitudes^5–7^, decrease in the temporal fluctuations of BOLD signal^8,9^, and suppressed functional connectivity (FC) both within and between resting state networks (RSNs)^2,6,9–13^. While most FC reduction has been reported in the default mode network (DMN), decreased FCs were also found in other RSNs, including the attention^2,6^, somatomotor^10^, salience^2^, visual^12^, and halamocortical^9,11^ networks.

Increasing evidence suggests that FC patterns are temporally dynamic rather than stationary throughout the length of the rs-fMRI scanning, even at different levels of consciousness^14–16^. A sliding-window technique followed by k-means clustering has been commonly employed to identify highly replicable sub-patterns of dynamic FC, named “brain states”^17,18^; where certain brain states can be associated with physiological and pathological brain conditions^19^. Comparing between conditions under general anesthesia (GA) and during wakefulness, distinct distributions of the identified sub-patterns of FC have been observed in animal studies^20,21^, as well as studies of healthy human subjects^2,22^. A similar approach (i.e. the sliding-window method and k-means clustering) was also applied in graph theory and energy-based analysis for the classification of two “clusters”; it was demonstrated that the brain condition during sevoflurane-induced anesthesia spent less time in an “integrated” state^12^ and a “low-energy” state^10^. Although conventional static FC analysis was able to show global reduction of FC under anesthesia, the dynamic FC approach has shown promising capability to detect nuanced changes in resting state FC patterns.

However, challenges remain for further explorations of the effects of sevoflurane on brain network connectivity. Given that different depths of anesthesia showed FC pattern variations^11,12^, it is critical to maximize the accuracy of sevoflurane levels during the rs-fMRI scanning, which is best achieved by endotracheal intubation. However, existing studies in the literature used sevoflurane concentrations ranging from 0.5 to 3%, and mostly administered with facemask or laryngeal mask in healthy volunteers^6,23,24^. To date, only one group has administered sevoflurane through endotracheal intubation during surgery for resection of pituitary microadenoma^8,9,25^. Additionally, previous studies focused on “healthy” brain architectures and utilized medically induced anesthesia as a model to investigate the pathology of disorders of consciousness. In this study, our goal was to investigate how sevoflurane affects dynamic functional communications in brains with existing neurological disorders. Potential findings might be able to provide transitional applications to clinical practice of monitoring functional activities in these patients.

## Methods

### Study design and participants

This prospective study received ethical approval by the Thomas Jefferson University Institutional Review Boards (IRBs), and all methods were performed in accordance with relevant guidelines and regulations. Written informed consent was obtained from all participants. To obtain stable volatile anesthesia state and to minimize interference with the routine clinical care of the participants, patients with drug-resistant epilepsy who were scheduled for laser interstitial thermal therapy (LITT) were selected for enrollment. LITT is routinely performed under general anesthesia in a magnetic resonance imaging (MRI) scanner with real-time temperature monitoring of the ablation and requires preoperative awake MRI for surgical planning. As such, this clinical scenario can be leveraged to acquire resting-state functional MRI (rs-fMRI) of the same subject both awake and under clinical general anesthesia (GA).

Nine adult patients were enrolled with the inclusion criteria: diagnosed with drug-resistant mesial temporal lobe epilepsy (mTLE), compliant with anti-seizure medication, and LITT candidates. Data of two patients were excluded during analysis due to poor data quality (one had excessive movements and one failed the quality check of the normalization processing). As a result, seven patients (mean ± SD age, 33.9 ± 12.0 years; 3 males; 4 females) were eventually involved in the following analysis.

### General anesthesia protocol and data acquisition

Each subject underwent rs-fMRI in two conditions: awake and under GA with an approximately 2 weeks interval in between. The first rs-fMRI scan (awake condition) was acquired preoperatively, and subjects were instructed to keep their eyes closed without falling asleep and relax without thinking about specific things. Straps and foam pads were used to minimize head movement. The second scan (GA condition) was acquired intraoperatively. Following a standard anesthetic protocol, a neuroanesthesiologist performed clinical pre-anesthetic preparation, induction of intravenous propofol (130–300 mg), and endotracheal intubation. Then, sevoflurane was administered through the endotracheal tube and maintained at 0.6–1.2 mean alveolar concentration (MAC). The hemoglobin level (Hgb) was measured via bloodwork. Approximately 15–20 min after induction to ensure propofol has worn off, the MRI for the GA condition was acquired, while the subject was stabilized under GA but before the LITT procedure. Physiologic variables were recorded at 1-min intervals and included heart rate (HR), mean arterial pressure (MAP), end-tidal carbon dioxide (ETCO2), peripheral capillary oxygen saturation (SpO2), respiratory rate, and body temperature. After the completion of LITT, patients were reversed with sugamadex (2–4 mg/kg), extubated as per routine, and were observed in a post-anesthetic care unit until fully recovered.

Both awake and GA rs-fMRI acquisitions were carried out on a 3.0T Philips Achieva MR scanner with an eight-channel head coil. The scanning protocol for both the conditions consisted of a T1-weighted sequence (field of view = 240 mm, voxel size = 1 × 1 × 1 mm^3^, matrix size = 352 × 352, TR = 7.5 ms, TE = 3.4 ms, slice thickness = 1 mm), followed by a 15 min rs-fMRI acquired with single-shot echo planar imaging (EPI) sequence (echo time = 30 ms, field of view = 230 mm, TR = 2000 ms, TE = 25 ms, flip angle 90°, voxel size = 2 × 2 × 3.5 mm^3^, matrix size = 128 × 128, slice thickness = 3.5 mm, 34 slices, a total of 450 volumes).

### Data preprocessing

Data preprocessing was performed using the CONN toolbox, version 20b^26^ (www.nitrc.org/projects/conn, RRID:SCR_009550). The first 10 volumes of each rs-fMRI scan were removed, and the remaining functional volumes were preprocessed with functional realignment, unwarping, and slice-timing correction. To maximally reduce the impact of head motion artifact^6^, outlier volumes were identified with a framewise displacement above 0.5mm or global BOLD signal changes above 20 z-scores^27^. The functional volumes and the corresponding anatomical images were segmented into 3 tissue regions (gray matter, white matter, and CSF) and normalized to the Montreal Neurological Institute (MNI) space at a resolution of 2 × 2 × 2 mm^3^. Then, the functional data was smoothed with a Gaussian kernel of 6mm full width half maximum (FWHM). A linear regression method was implemented to regress out confounding variables, including the white matter, CSF, head motion parameters, and the identified outliers. The residual data were band-pass filtered between 0.01 and 0.15 Hz and linear detrended.

**Figure 1:**
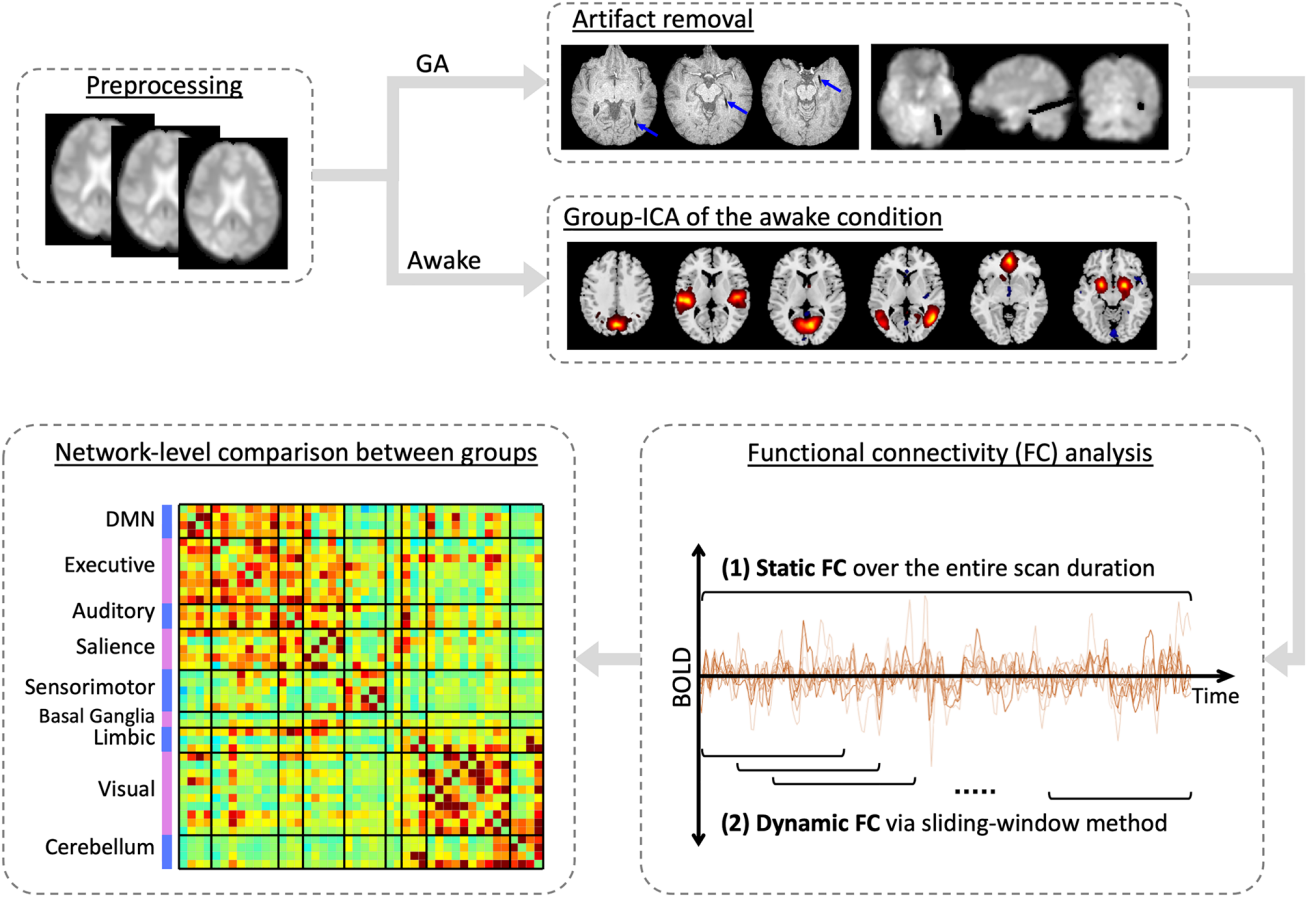
Pipeline of functional connectivity data analysis. The blue arrows point at the artifact around a laser probe on MR images during general anesthesia (GA). Group-ICA of the awake condition, followed by manual selections, resulted in multiple independent components (six examples displayed).

In five patients, the intraoperative rs-fMRI was acquired after the placement of a laser probe, which was associated with artifact around the laser probe on the MR images. The artifact region was manually segmented on each individual’s corresponding T1 image using ITK-SNAP. To minimize the effects of the artifact on the FC analysis, the segmentation was expanded just beyond the artifact by a few voxels; and then voxels of the artifact region were excluded from both the GA, as well as the awake, rs-fMRI scans (Fig. 1). The resulting data was used in the following analysis.

### Group independent component analysis

To avoid potential anatomical bias from atlases, we performed group-level spatial independent component analysis (group-ICA), a data-driven method to identify regions of interest (ROIs) that fit better to the cohort. These regions are referred to as independent components (ICs). To overcome the issue of reduced amplitudes of BOLD signals affected by anesthetic agents^6,9,28^, group-ICA was executed using only the awake rs-fMRI data of all subjects.

The optimal number of ICs was estimated using the minimum description length (MDL) algorithm. A two-step principal component analysis (PCA) was applied for subject-specific data reduction followed by group-level data reduction. Infomax ICA was repeated 20 times in ICASSO to assess its reliability. The spatial maps were obtained with cutoff values of mean ± 4 standard deviations (SD)^17^. Then, the estimated ICs were manually inspected by three authors (JM, MA, and CW) to identify signal components from noise components, based on a previously described method. Briefly, signal components have peak activation in gray matter, the predominant power in low frequency range (0.01–0.1 Hz), and fewer fluctuations throughout the scanning session^29^. The selected signal components were categorized into nine well-described RSNs based on spatial distributions^4^ and were included into the following FC analyses. The RSNs included the DMN, executive, auditory, salience, sensorimotor, basal ganglia, limbic, visual, and cerebellum networks.

The MATLAB-based GIFT toolbox (https://trendscenter.org/software/gift) was used for all steps of group-ICA and FC analyses^30^.

### Static and dynamic functional connectivity analysis

Functional connectivity was estimated through an ROI-to-ROI approach, which creates a FC matrix from temporal correlation analysis between signal components within each pair of ROIs (Fig. 1). A multivariate analysis of covariance (MANCOVA) was applied to identify individual-level covariables (including age, gender, Hgb, HR, MAP, ETCO2, SpO2, respiratory rate, and body temperature) that had significant effects on the FC matrix^17^.

Static FC was estimated by correlating the entire timeseries (440 volumes) of each rs-fMRI session, resulting in 14 static-FC matrices (a matrix during wakefulness and a matrix under GA for each subject). The mean static-FC matrices of the awake and the GA conditions were then computed.

Temporal dynamic FC was estimated by applying a sliding window approach followed by k-means clustering^18^. A tapered window was created by convolving a rectangle of 30 TRs (60s) with a 3 TR Gaussian kernel and sliding it by 1 TR (2s) step size. This resulted in 410 windows for each rs-fMRI session. The timeseries of each window was used to estimate a window-FC matrix. To improve the sparsity and comparability of the matrices obtained from short time segments, window-FC matrices were further processed using the graphical LASSO algorithm with a L1 penalty followed by the Fisher transformation^18,31^. After removal of the significant covariables determined by MANCOVA, we applied k-means clustering to the entire cohort’s window-FC matrics. This clustering method classified matrices based on similarities in FC patterns. Each resulting cluster is referred to as a brain state (or state) representing the highly repeated FC sub-patterns during rs-fMRI. The estimated optimal number of clusters (K) varied between 2 and 7 between algorithms (including the elbow method, gap statistic, BIC/AIC criteria, silhouette statistic, Dunns, Daves Bouldin, and Ray Turi metrics). We therefore visually assessed the similarity and differences of centroids across this cluster range (Supplementary Fig. 1). It was thereby determined that four clusters provided the best representative brain states. To ensure the accuracy and reproducibility of clustering, k-means clustering was repeated 500 times using city-block distance^18^. Then, the mean FC (or the centroids) of each brain state was computed separately for the entire cohort, the awake condition, and the GA condition. Quantitative metrics of the dynamic FC analysis were also calculated for the awake and the GA conditions, including (1) the *fraction time*, indicating the percentage of the entire scanning duration spent in each dynamic state; (2) the *mean dwell time*, the duration for which a certain dynamic state tends to remain; and (3) the *number of state transitions*, with higher values indicating more fluctuations of dynamic states during each session.

### Statistical analysis

Statistical comparisons were performed on the network level of FC matrices between the awake and the GA conditions using Wilcoxon rank sum test followed by the Bonferroni-Holm correction for multiple comparisons (significance level < 0.05). The same tests were applied to the static FCs, as well as brain state 2 and 3 from the dynamic FC analysis. Given that brain state 1 and 4 were found exclusively present during GA and wakefulness, respectively, and thus were not statistically compared between these two conditions. The metrics of the dynamic FC analysis (the fraction time, mean dwell time, and the number of state transitions) were compared between two groups using student t-test with FDR correction (significance level < 0.05).

## Results

### Group independent component analysis

**Figure 2:**
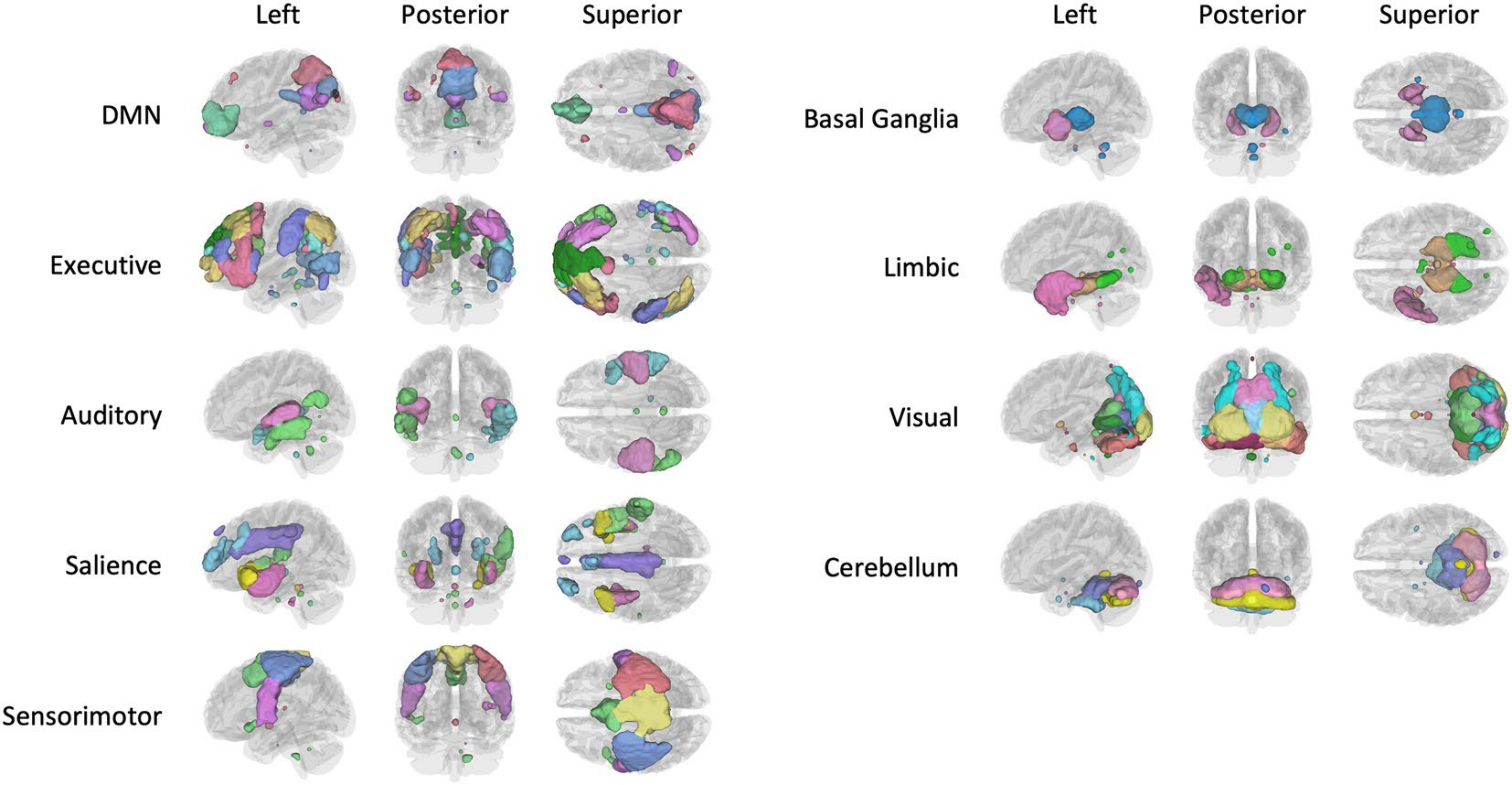
Forty-four selected signal components grouped into nine resting state networks (RSNs), consisting of the default mode network (DMN), executive, auditory, salience, sensorimotor, basal ganglia, limbic, visual, and cerebellum networks. Different colors within each network are used for visualization, each representing an independent component (IC).

The estimated optimal number of ICs for the awake rs-fMRI data was 67 (SD = 10). Out of the 67 ICs identified by group-ICA, 44 signal components were manually identified and sorted into nine RSNs, including the DMN, executive networks, auditory networks, salience networks, sensorimotor networks, basal ganglia networks, limbic networks, visual networks, and cerebellum networks (Fig. 2). The peak activation coordinates in MNI space are listed in Supplementary Table 1.

### Static and dynamic functional connectivity

**Figure 3:**
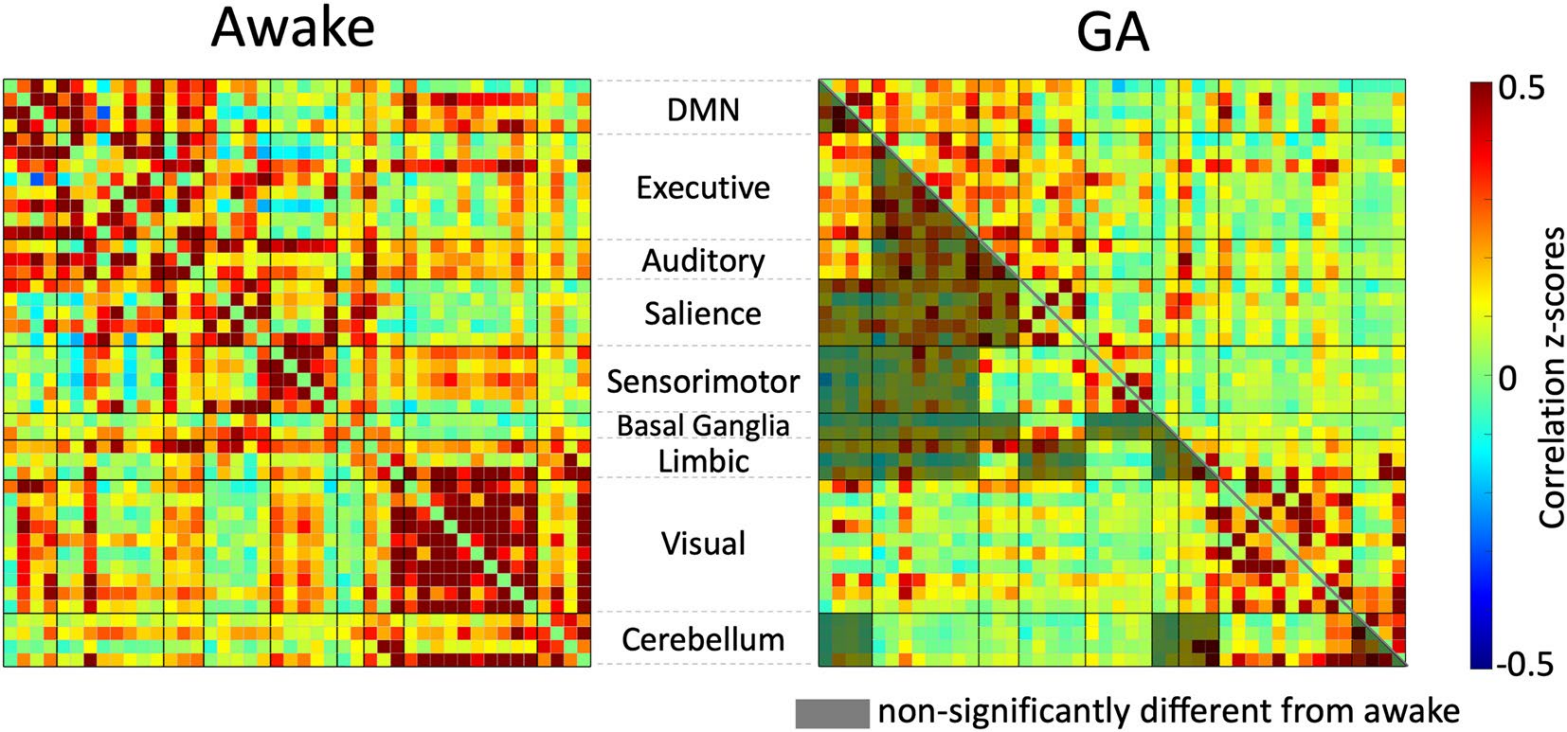
Static functional connectivity (sFC) of the awake and the general anesthesia (GA) conditions. In the lower triangular part of the GA sFC matrix, the shaded areas label the non-significantly different FCs from the awake condition, while the non-shaded areas of the lower triangular part represent significant FC differences between the two conditions (significance level *p* < 0.05 after Bonferroni correction).

The MANCOVA univariate test revealed that the overall FCs were significantly affected by age, gender, and Hgb levels, which were then regressed out for the FCs from each subject. The static FC matrices of the awake and the GA conditions were shown in Fig. 3. On the network level, 22 out of the 45 pairs of networks (both within and between RSNs) showed significantly reduced connectivity transitioning from awake to GA condition. Specifically, decreased intra-network FCs were observed within the salience, sensorimotor and visual networks; decreased inter-network FCs were mostly associated to the auditory, sensorimotor, visual, and cerebellum networks.

**Figure 4:**
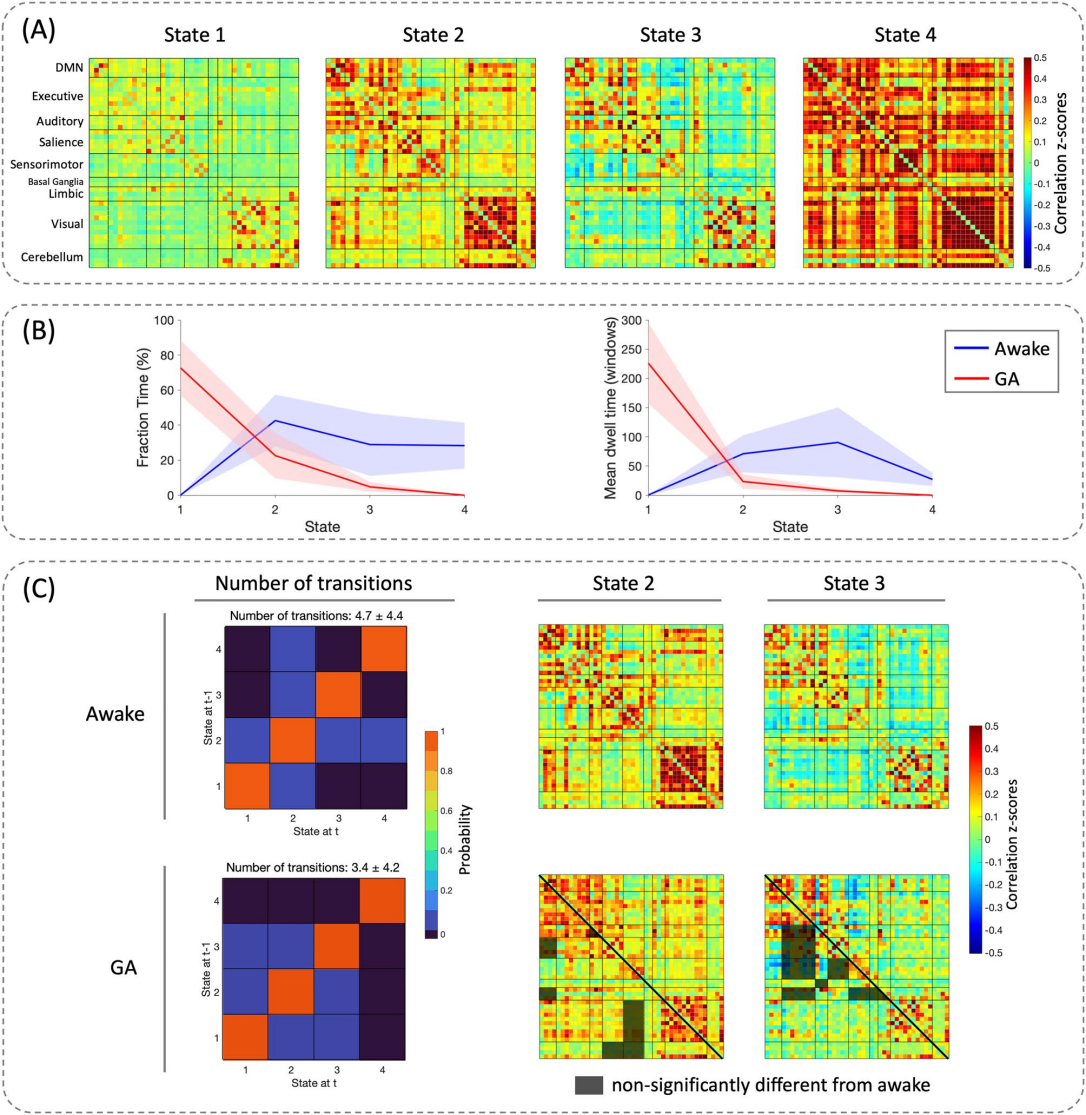
Dynamic functional connectivity (dFC) and statistical results. (**A**) Mean FCs of the four brain states resulted from k-means clustering. (**B**) The fraction time and the mean dwell time of each brain state compared between the awake and the general anesthesia (GA) conditions. The shaded region represents the estimated probability. (**C**) The number of state transitions and the mean FCs of State 2 and 3 for the awake and the GA conditions. In the lower triangular part of the GA FC matrices, the shaded areas label the network-level FCs that were not significantly different from wakefulness (significant level *p* < 0.05 after Bonferroni correction).

From the dynamic FC analysis, four brain states were clustered from the entire cohort and the mean matrix of each brain state was shown in Fig. 4. The number of transitions between brain states was decreased under GA (3.4 ± 4.2) vs. awake (4.7 ± 4.4). Regarding the fraction time, State 4 was only observed during awake, whereas State 1 was mostly seen under GA; State 2 and 3 were shared by both conditions, specifically 72% of the window-FCs during wakefulness and 27% of those under GA were grouped into these two brain states. Moreover, FC patterns during wakefulness moved across three brain states (State 2, 3, and 4), while GA mainly expressed a single FC pattern (State 1) with reduced time spent in State 2 and 3. Regarding the mean dwell time, GA showed preference to stay in State 1, whereas awake condition showed relatively even distribution among State 2, 3, and 4. Furthermore, comparing between the awake and the GA conditions during State 2 and 3 revealed significant differences on a network level of FCs, both within and in-between RSNs (Fig. 4C).

## Discussion

Brain network FC analyses help to reveal the effects of sevoflurane-induced GA. This rs-fMRI study is the first to investigate static and dynamic FC changes during GA in patients with refractory mTLE. The results demonstrate distinct FC patterns and fewer transitions between brain states during GA, that are in line with previous studies. Specifically, a brain state with high synchronization was the characteristic FC pattern of wakefulness, while a brain state with the least connectivity was characteristic of the GA condition; wakefulness fluctuated among multiple brain states, but GA suppressed such fluctuations which led to a single leading brain state. We further looked into each of the shared brain states and compared the awake and GA conditions. Different networks were affected by GA in different brain states, which provides additional support for the previously proposed hierarchical effects of GA on a more granular level.

The static FC analysis demonstrates significant decrease of FC in some RSNs on top of a uniform reduction of the entire brain connectivity, which was in line with previous findings on sevoflurane-, as well as propofol-, induced GA^2,6,9^. Despite variant findings among previous research, many studies have reported decreased FC predominantly within the DMN^2,6,9–12^ and between the thalamus and frontal cortex^6,9,12,24,32^, but demonstrated relatively preserved connectivity among the sensory networks^2,13,33^. Partially aligned with previous findings, our cohort presented significantly decreased FC within the salience network, between DMN and executive, and between the limbic and sensorimotor network. However, no significant reduction within the DMN was detected under GA, which was also reported by Venkatraghavan and colleagues^32^. Additionally, our cohort demonstrated significantly decreased FC related to the sensory networks, including the auditory, sensorimotor, and visual networks. Interestingly, participants in our study had their eyes closed in both the awake and the GA conditions, yet significant reduced FC was detected both within and between the visual network and other RSNs. Although less commonly reported, a few previous studies also demonstrated similar findings of the sensory networks, namely the somatosensory network^10,11,34^ and the visual network^11,12^. Our static FC patterns of GA also detected significantly suppressed FC between the cerebellum network and other RSNs (except for the DMN, basal ganglia, and limbic networks). Only a limited number of studies have considered the cerebellum network and demonstrated similar findings of decreased FC in both sevoflurane-induced^11,34,35^ and propofol-induced GA studies^14^, which underscore the need for further research to elucidate the role of cerebellum in consciousness.

Our dynamic FC findings were in line with previous GA studies in healthy subjects and monkeys regardless of how many brain states were chosen^2,20^. During wakefulness, the dynamic FCs had higher rates of transitions between brain states and were roughly evenly distributed among State 2, 3, and 4—patterns that were characterized by higher correlations among sensory, executive, and default mode networks. State 4, characterized by highly synchronized brain activity within the auditory, sensorimotor, and visual networks, was exclusively observed during awake. On the other hand, GA showed a reduced number of transitions, accompanied by a single predominant brain state (State 1), which clearly had the highest fraction time and the longest mean dwell time than other states. Meanwhile, State 1, characterized by the lowest correlation among all the networks, was almost exclusively found under GA. Additionally, the two brain states (State 2 and 3) that were shared by the awake and the GA conditions were characterized by the presence of anti-synchronization between networks; their overall connectivity strengths were somewhere in between State 1 and 4, which seemed further to illustrate the GA-induced “shift” between brain states.

Therefore, we further investigated each of the shared brain states and found statistically significant differences in the majority of network FCs comparing the awake and the GA conditions. This may be explained by the universal effects of GA on the entire brain. However, interestingly, the spared network connectivity (i.e. the non-significantly different correlations) was distinct between the two brain states: the executive and limbic networks were mainly conserved in State 3 and the sensorimotor network in State 2. Previous studies have proposed hierarchical effects of anesthesia from multiple aspects, namely, the spatial influence on specific brain networks, the temporal influence on fluctuations of brain connectivity^2,7,8^, and a preferential modulation of the feedback/feedforward pathway^36^. Although more studies are warranted to explain our observation in these shared brain states, such findings could provide new insights into the hierarchical effects of GA on a more granular level.

This study had several strengths, such as the use of endotracheal intubation for administering sevoflurane, the long duration of rs-fMRI scans, and our relatively large sample size in patient populations. Given that FC patterns and dynamic FC transitions are affected by the depth of anesthesia^2,11,12,20^, it is especially critical to stabilize the sevoflurane level during rs-fMRI acquisition in dynamic FC analysis to minimize its effects on window-FC patterns. Intubation via endotracheal tube can provide more accurate sevoflurane administration than laryngeal mask and facemask, which were commonly used in previous studies. In the present study, the sevoflurane level was consistently maintained within a variation of approximately 0.2 MAC for each individual subject, despite the varying levels of sevoflurane required across patients (0.6–1.2 MAC). Additionally, the test–retest reliability of FC analysis has been proven to be highly affected by rs-fMRI scan length. Generally, with a smaller sample size and the involvement of intra-network FC analysis, the scan duration during awake should be at least 15 min^28,37,38^. Therefore, a possible explanation for the inconsistent or controversial findings in studies to date could be caused by the short scan time. Most previous studies acquired 8–10 min of rs-fMRI, which may lead to relatively reduced test–retest reliability across FC studies. In the present study, a 15-min rs-fMRI scan was collected to increase the reliability of FC findings. To the best of our knowledge, our study involved the largest cohort of patients with endotracheal intubation (n = 7) and is the first study that examined the effects of GA on patients with neurological disorders.

Several limitations were also acknowledged in this study. First, as we aim to generalize previous findings about the effects of sevoflurane-induced anesthesia to a patient population with neurological diseases, the subject population was limited to patients with drug-resistant epilepsy undergoing LITT due to ethical reasons. Second, our earlier study has revealed that sevoflurane-induced GA may modulate the brain activity and the cerebrovascular components differently^7^. However, we could not regress out the cerebrovascular changes as confounding variables in the present study. Instead, to maximize the accuracy of FC analysis, MANCOVA was executed to identify the significant confounding variables (i.e. age, gender, and MAP), which were then removed from FC analysis. Third, although all subjects attained a brain state of general anesthesia in a clinical surgery setting, the varying concentrations of sevoflurane required across subjects (0.6–1.2 MAC) due to inter-individual pharmacokinetic variation may act as a confounding variable.

In conclusion, dynamic FC patterns during GA were characteristic with a single brain state that is distinct from those in the awake condition. Transitioning from wakefulness to GA condition, both the strengths of synchronization and the number of transitions between brain states were suppressed. Dynamic FC analysis can be used to investigate nuanced effects of sevoflurane both spatially and temporally. However, it is critical to take into consideration the accuracy of sevoflurane levels and the duration of rs-fMRI scans, as they can affect FC patterns and the test–retest reliability of FC analysis. Future studies are warranted to further investigate the hierarchical effects of sevoflurane on network functional connectivity.

### Supplementary Information

Supplementary Information.

## Data Availability

The data that support the findings of this study are available on request from the corresponding author, JM.
