# Supplementary material for: Characteristic dynamic functional connectivity during sevoflurane-induced general anesthesia
Source: Sci Rep. 2023 Nov 29;13:21014. doi: 10.1038/s41598-023-43832-1 (PMC10687074; doi:10.1038/s41598-023-43832-1)
Supplement: Supplementary file 1 — Supplementary Information. [file 41598_2023_43832_MOESM1_ESM.pdf]

Supplementary Figure 1. The centroids of clusters, using k-means clustering, across K=2-7 for visual assessments of the optimal cluster number of this study.

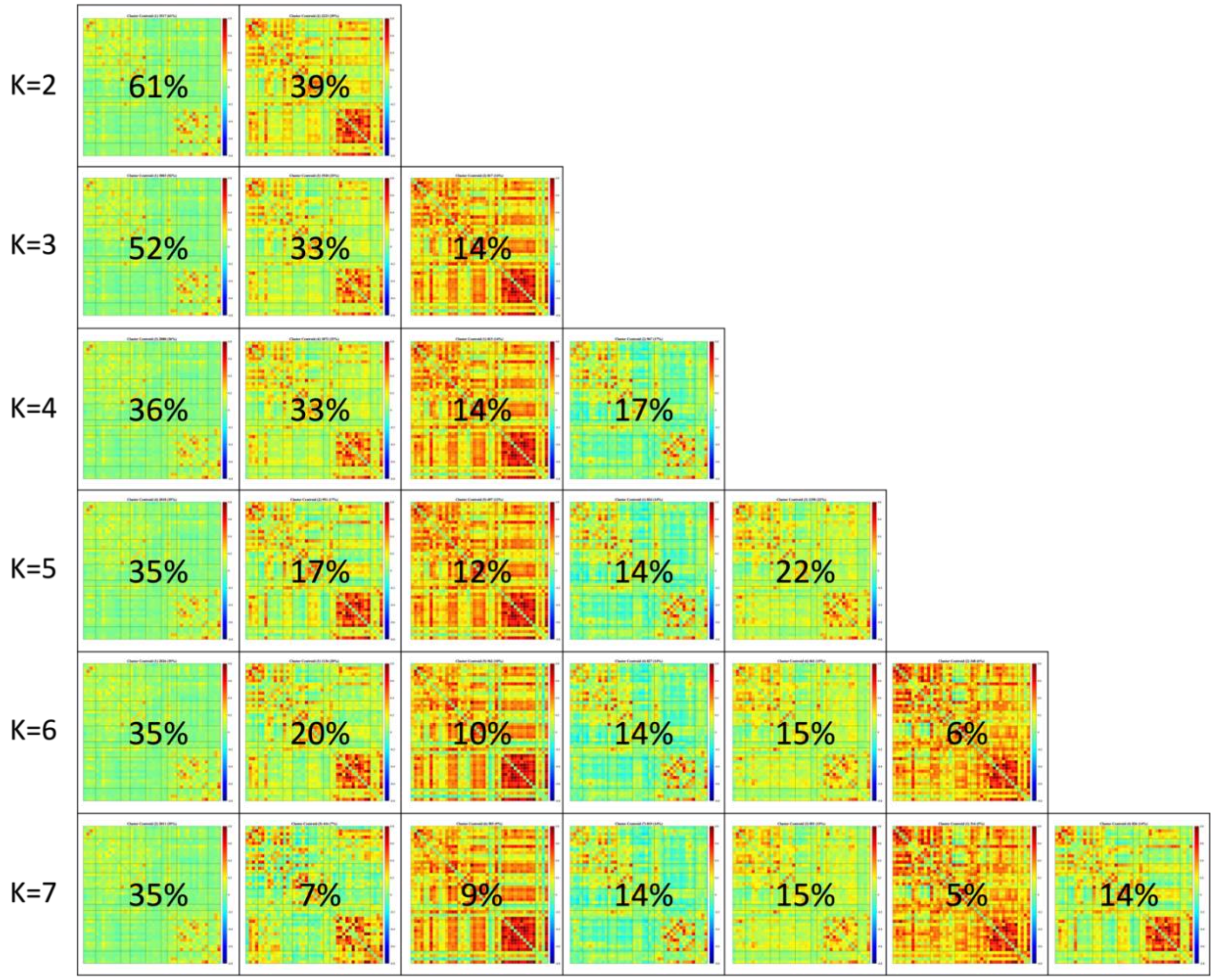

Supplementary Table 1. The peak coordinates in MNI space of the selected independent components (ICs) and the resting state networks (RSN) of each IC.

IC #	Peak Coordinates (mm) (x, y, z)	RSNs
2	(-36.5, 19.5, 62.5)	Sensorimotor
4	(-49.5, -6.5, 22.5)	Sensorimotor
5	(9.5, -53.5, 6.5)	Visual
6	(0.5, 52.5, -7.5)	DMN
7	(12.5, -34.5, -43.5)	Cerebellum
10	(31.5, -13.5, 68.5)	Sensorimotor
11	(1.5, -20.5, 72.5)	Sensorimotor
13	(-44.5, -25.5, 7.5)	Auditory
14	(0.5, -72.5, 36.5)	DMN
15	(-23.5, -91.5, 1.5)	Visual
16	(8.5, -68.5, 5.5)	Visual
18	(-2.5, -84.5, 21.5)	Visual
20	(0.5, 10.5, 30.5)	Salience
21	(0.5, -60.5, 26.5)	DMN
22	(-39.5, -2.5, -16.5)	Salience
24	(0.5, -20.5, 0.5)	Basal Ganglia
25	(0.5, -61.5, 46.5)	DMN
27	(2.5, 43.5, 49.5)	Executive
29	(-2.5, -86.5, -15.5)	Visual
30	(3.5, -71.5, -22.5)	Cerebellum
33	(9.5, -51.5, -0.5)	Visual
34	(26.5, 30.5, 52.5)	Executive
35	(10.5, -39.5, -18.5)	Cerebellum
36	(5.5, -88.5, 2.5)	Visual
37	(22.5, 1.5, -13.5)	Basal Ganglia
39	(50.5, -60.5, 4.5)	Executive
40	(-25.5, -81.5, 20.5)	Visual
41	(-52.5, 6.5, -14.5)	Limbic
43	(40.5, -58.5, -23.5)	Visual
44	(0.5, -4.5, 60.5)	Sensorimotor
46	(-44.5, -38.5, 51.5)	Executive
47	(19.5, -81.5, -17.5)	Visual
48	(19.5, -26.5, -16.5)	Limbic
49	(-49.5, 20.5, 0.5)	Executive
51	(-61.5, -23.5, -10.5)	Auditory
52	(26.5, -48.5, -10.5)	Limbic
53	(21.5, -67.5, -16.5)	Cerebellum
54	(-19.5, 29.5, 55.5)	Executive
56	(48.5, 14.5, 28.5)	Executive
58	(-42.5, 13.5, -8.5)	Salience
59	(0.5, -22.5, -4.5)	Salience
60	(56.5, -30.5, -2.5)	Auditory
61	(39.5, -3.5, -15.5)	Salience
63	(53.5, -53.5, 18.5)	Executive
